# MRI assessment of cardiomyopathy in Taiwan Chinese late-onset Fabry mutation (IVS4+919G>A)

**DOI:** 10.1186/1532-429X-17-S1-P276

**Published:** 2015-02-03

**Authors:** Yu-Pin Chang, Jyh-wen Chai, Yi-Ying Wu, Yun-Ching Fu, John Wang, Clayton Chi-Chang Chen

**Affiliations:** Radiology, Taichung Veterans General Hospital, Taichung, Taiwan; Pediatrics, Taichung Veterans General Hospital, Taichung, Taiwan; Pathology, Taichung Veterans General Hospital, Taichung, Taiwan

## Background

Fabry disease is a rare X-linked disorder characterized by deficiency ofα-galactosidase A, leading to progressive accumulation of glycosphingolipid in various organs, including the heart. Recently, several later-onset phenotypes of Fabry disease with residual enzyme activity have been identified. In Taiwan, several recent studies pointed out a surprisingly high incidence of a later onset Fabry mutation (IVS4+919G>A) and there is a lack of information about its cardiac MR appearance in the literature. We aim to present the cardiac MR appearances of this subtype of Fabry disease and compare them with the classic type.

## Methods

A total of 12 patients (9 males and 3 females) were enrolled in this study. They underwent endomyocardial biopsy as well as MR study from July 2013 to September 2014 and were proved to be Fabry mutation (IVS4+919G>A). We recorded the location of delayed myocardial enhancement according to modified AHA 16-segment model and measured left ventricular (LV) wall thickness of each segment in short axis view at the end diastole. We categorized these patients into three groups (Group I, II, III) according to their myocardial thickness(all segments<12mm, at least one segment 12~16mm, at least one segment>16mm, respectively ). Among the all patients, we used Χ square test to evaluate if there was significant difference in presence of delayed enhancement according to different myocardial wall thickness.

## Results

There were 12 patients with 192 myocardial segments (male: 144 segments, female: 48 segments) under evaluation. There were 4 patients in Group III with 22 segments>16mm, 4 patients in Group II with 5 segments in range of 13~16mm. The remained 4 patients were in Group I. in Group III were. In Group III, there were 15 segments>16mm involving the septum and 13 segments in range of 13~16mm. Besides, there were 37 segments with delayed enhancement in Group III, with 14 in posteriolateral wall, 13 in anterior wall and 10 involving the septum. In group I and II, two patients with 4 segments demonstrated delayed enhancement and the 4 segments (segment 4, 10, 6, 11) are all in the normal range of thickness(<12mm). In the Χ square test evaluation, there was significant difference in presence of delayed enhancement dependent on various myocardial wall thickness (P<0.05).

## Conclusions

In our study, patients with Fabry variant demonstrated some different patterns of delayed myocardial enhancement and wall thickness in cardiac MR as compared with classic Fabry disease.

**Figure 1:**
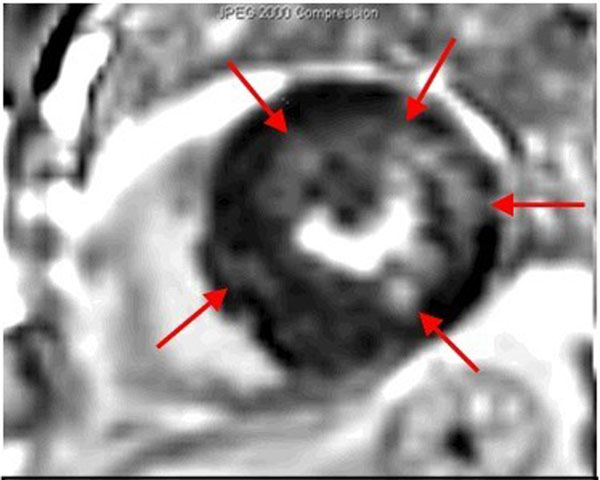
A 68 y/o male with Fabry variant demonstrates delayed myocardial enhancement and wall thickening (>16mm) in multiple LV segment, including segment 7,8, 10, 11 and 12 (red arrows). (Other segments were also involved, but not shown in this image.)

**Figure 2:**
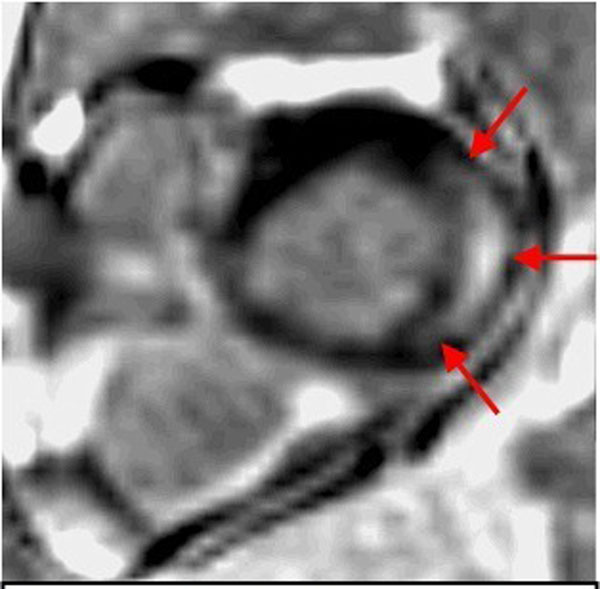
A 55 y/o male with Fabry variant shows delayed myocardial enhancement in LV segment 10, 11 and 12 (red arrows). These segments were only 12~13mm in thickness.

